# Clinical characteristics and associated factors of cardiovascular involvement in patients with relapsing polychondritis

**DOI:** 10.3389/fmed.2026.1877659

**Published:** 2026-07-31

**Authors:** Tianyan Shi, Qi-Ming Liu, Xiao-Hong Wen, Ying-Fang Zhang, Yue-Wu Lu, Juan Meng

**Affiliations:** Beijing Chaoyang Hospital, Capital Medical University, Beijing, China

**Keywords:** associated factors, cardiac involvement, clinical characteristics, low-density lipoprotein, relapsing polychondritis

## Abstract

**Aims:**

To analyze the clinical characteristics and identify factors associated with cardiovascular involvement in patients with relapsing polychondritis (RP).

**Methods:**

A total of 105 RP patients were enrolled and divided into cardiac and non-cardiac groups. Demographic, clinical, and serological data were collected and analyzed comparatively. Univariate and Firth's logistic regression analyses were performed to identify independent factors associated with cardiac involvement. Hierarchical clustering was applied to explore phenotypic patterns.

**Results:**

Among the RP cohort, 29.5% (31/105) exhibited cardiovascular involvement, with a male-to-female ratio of 16:15 and a mean age of 57.87 ± 12.06 years. Manifestations included aortic root dilatation (11.4%), conduction abnormalities (8.6%), myocarditis (7.6%), pericarditis (5.7%), and valvular dysfunction (3.8%). Patients with cardiac involvement exhibited a significantly lower prevalence of ocular involvement (12.9 vs. 36.5%; *P* = 0.016) and a non-significant trend toward lower frequency of costochondritis (9.7 vs. 24.3%; *P* = 0.087). Notably, low-density lipoprotein (LDL) levels were significantly lower in the cardiac involvement group [2.60(1.00, 4.37) vs. 2.84(1.45, 5.30) mmol/L; *P* = 0.041]. Firth's logistic regression identified the ocular involvement (OR: 0.22, 95% CI: 0.06–0.78; *P* = 0.010), costochondritis (OR: 0.26, 95% CI: 0.07–0.99; *P* = 0.029), and LDL levels (OR: 0.51, 95% CI: 0.27–0.93; *P* = 0.017) as independent factors associated with cardiac involvement. Clustering analysis identified five phenotypic groups, with the “tracheobronchi-cardiac” cluster showing the lowest LDL levels and the smallest proportions of ocular and costal cartilage involvement.

**Conclusions:**

Cardiac involvement in RP is not rare and is independently associated with lower LDL levels and reduced frequencies of ocular and costal cartilage involvement. These findings may help identify RP patients at risk for cardiac manifestations and suggest potential protective roles of certain clinical features.

## Introduction

Relapsing polychondritis (RP) is a rare systemic autoimmune disease, with an estimated annual incidence of 0.71–3.5 cases per million (1, 2). It predominantly affects individuals in their fourth to fifth decades of life. The disease primarily targets cartilage-related connective tissues, including the ears, nose, trachea, and bronchi. In addition to these classic cartilaginous involvement, RP exhibits a diverse spectrum of systemic manifestations, encompassing dermatological, renal, neurological, and vascular complications (3). The multisystemic nature of RP frequently poses diagnostic challenges, leading to an average diagnostic delay of 1.06 years after symptom onset (4).

Mortality in RP varies and is influenced by the severity of respiratory and cardiovascular complications, as well as infections potentially related to immunosuppressive therapy. The rarity and relapsing-remitting nature of RP challenge the acquisition of accurate epidemiological data on cardiac involvement. Current knowledge is primarily derived from small case series and literature reviews (5). Although often underestimated, cardiovascular involvement can lead to severe complications and a poorer prognosis, sometimes necessitating surgical intervention (6).

This study aims to characterize the clinical features and identify factors associated with cardiac involvement in RP, with the ultimate goal of facilitating early detection and improving prognosis.

## Methods

### Patients

We conducted a retrospective review of clinical information from 146 patients diagnosed with RP at Beijing Chaoyang Hospital between January 2013 and December 2024. The diagnosis of RP was established based on the criteria proposed by McAdam et al. (2) or Michet CJ et al. (7). Patients with incomplete clinical data or cardiovascular involvement attributable to other etiologies were excluded from the analysis. This study was approved by the Ethics Committee of Beijing Chaoyang Hospital and was conducted in accordance with the principles of the Declaration of Helsinki. Informed consent was waived by the Institutional Review Board for this retrospective study.

### Disease assessment

Clinical data of all patients were extracted from medical records, including demographic characteristics (age, sex, smoking history, and alcohol use), disease duration, clinical signs and manifestations, organ involvement, cardiovascular risk factors, laboratory parameters, and treatment regimens. Laboratory data collected comprised routine blood tests, assessments of liver and kidney function, lipid profiles, levels of immunoglobulins and complement, erythrocyte sedimentation rate (ESR), C-reactive protein (CRP), rheumatoid factor (RF) titers, anti-cyclic citrullinated peptide antibodies, anti-nuclear antibodies (ANA), anti-extractable nuclear antigen (ENA) profiles, and anti-neutrophil cytoplasmic antibodies (ANCA).

Cardiovascular involvement was comprehensively assessed and confirmed through a combination of blood tests, electrocardiography, echocardiography, and/or cardiovascular magnetic resonance (CMR). Specific cardiovascular manifestations assessed included valvular disease, aortic disease, pericarditis, myocarditis, and arrhythmia. All instances of organ involvement were verified based on clinical presentation and corroborated by ancillary examinations.

In transthoracic echocardiography, aortic root dilatation is defined as an aortic root diameter ≥40 mm. Moderate-to-severe valvular dysfunction is defined using the ASE criteria: for regurgitation, vena contracta ≥0.4 cm (mitral), ≥0.3 cm (aortic/pulmonary), or ≥0.5 cm (tricuspid), with pressure half-time < 200 ms (aortic) or < 100 ms (pulmonary) and hepatic vein reversal (tricuspid); for stenosis, peak velocity ≥3.0 m/s (aortic/pulmonary), mean gradient ≥20 mmHg (aortic/pulmonary) or ≥5 mmHg (mitral/tricuspid), and valve area ≤ 1.5 cm^2^. Pericardial effusion is determined as an echo-free space in diastole ≥10 mm.

Myocardial inflammation on CMR is defined according to the updated Lake Louise Criteria (8): at least one T1-based criterion (increased myocardial T1 relaxation times, extracellular volume fraction, or late gadolinium enhancement) together with at least one T2-based criterion (increased myocardial T2 relaxation times, visible myocardial edema, or increased T2 signal intensity ratio).

### Statistical analysis

Continuous data were expressed as the median (range) or mean ± standard deviation, depending on their distribution. Categorical data were presented as frequencies and percentages. Comparisons between groups were performed using the Chi-square test (or Fisher's exact test for small samples) for categorical variables, and Student's *t*-test or Mann–Whitney *U*-test for continuous variables. Spearman or Pearson correlation analysis was used to evaluate associations between cardiovascular involvement and clinical parameters, as well as correlations among the clinical parameters themselves. Univariate analysis was used to screen potential factors, and those with a *P*-value < 0.1 were assessed for multicollinearity using the variance inflation factor (VIF), with a threshold of 5. Subsequently, the potential factors were included in Firth's logistic regression to identify independent risk factors, with outcomes detailed as odds ratios (ORs) and corresponding 95% confidence intervals (CIs). A two-sided *P*-value < 0.05 was considered statistically significant. The statistical analyses were performed using SPSS version 21.0 (IBM Corp., Armonk, NY, USA) and Python (version 3.8) along with its packages.

## Results

### Baseline characteristics and laboratory findings of the RP cohort

This study enrolled 105 of 146 hospitalized RP patients (71.9%) after applying the predefined inclusion and exclusion criteria. The cohort included 51 female and 54 male patients, with a female-to-male ratio of 0.94. The mean age of the participants was 55.57 ± 11.34 years.

The systemic involvement at diagnosis was detailed as follows: tracheobronchial involvement (*n* = 85, 81.0%), external ear involvement (*n* = 54, 51.4%), nasal involvement (*n* = 38, 36.2%), arthritis (*n* = 31, 29.5%), ocular involvement (*n* = 31, 29.5%), costal cartilage involvement (*n* = 21, 20.0%), hearing loss (*n* = 14, 13.3%), thyroid cartilage involvement (*n* = 8, 7.6%), cutaneous lesions (*n* = 8, 7.6%), and peripheral nervous system involvement (*n* = 2, 1.9%).

The study population comprised 63 non-smokers (60.0%) and 86 non-drinkers (81.9%). Regarding comorbidities, hypertension was observed in 20 patients (19.0%), diabetes mellitus in 27 patients (25.7%), and hyperlipidemia in 31 patients (29.5%). Concomitant connective tissue diseases (CTDs) were documented in 12 patients (11.4%), including primary Sjögren's syndrome (pSS) (*n* = 5), ANCA-associated vasculitis (AAV) (*n* = 2), systemic lupus erythematosus (SLE) (*n* = 2), rheumatoid arthritis (RA) (*n* = 1), Behçet's disease (MAGIC syndrome, *n* = 1), and ankylosing spondylitis (*n* = 1).

Detailed laboratory test results are summarized in Table 1. The median duration from the patient's initial visit for RP to the last follow-up was 27 months (range, 3–146 months). During this period, three patients died from severe pulmonary infections secondary to progressive tracheobronchial cartilage destruction. Two patients underwent percutaneous coronary intervention with stent implantation, which was not clearly related to RP (both patients had hypertension and type 2 diabetes mellitus). No patient experienced sudden cardiac death or heart failure with reduced ejection fraction.

**Table 1:** Baseline clinical characteristics for patients with relapsing polychondritis (*n* = 105).

Clinical characteristics	Outcomes
Age (ys)	55.57 ± 11.34
Gender (F/M)	51/54
WBC (^*^10^9^/L)	7.91 (0.81, 26.09)
NEU%	69.5 (23.5, 96.0)
LY%	22.5 (0.9, 61.7)
NLR	3.05 (0.38, 99.44)
PLR	163.07 (53.02, 1004.02)
TC (mmol/L)	4.41 (2.23, 7.73)
LDL (mmol/L)	2.79 (1.00, 5.30)
HDL (mmol/L)	1.17 (0.57, 3.38)
TG (mmol/L)	0.98 (0.37, 3.31)
ALB (g/L)	37.2 (23.0, 53.8)
IgG (mg/dl)	1210.0 (485.0, 2420.0)
IgA (mg/dl)	254.0 (61.7, 634.0)
IgM (mg/dl)	87.4 (27.7, 642.0)
C3 (mg/dl)	111.0 (47.6, 182.0)
C4 (mg/dl)	26.5 (11.0, 66.5)
CRP (mg/dl)	1.90 (0.10, 31.60)
ESR (mm/h)	29.0 (2.0, 120.0)
CAR	0.51 (0.03, 9.52)
Concomitant CTD	11.4%
Hypertension	19.0%
Diabetes mellitus	25.7%
Hyperlipidemia	29.5%
Smoking history	40.0%
Drinking history	18.1%
Cutaneous lesions	7.6%
Costal cartilage involvement	20.0%
Ocular involvement	29.5%
Arthritis	29.5%
Nasal involvement	36.2%
External ear involvement	51.4%
Tracheobronchial involvement	81.0%

WBC, white blood cell; NEU, neutrophil; LY, lymphocyte; NLR, neutrophil-to-lymphocyte ratio; PLR, platelet-to-lymphocyte ratio; TC, total cholesterol; LDL, low-density lipoprotein cholesterol; HDL, high-density lipoprotein cholesterol; TG, triglycerides; ALB, albumin; Ig, immunoglobulin; C3, complement 3; C4, complement 4; CRP, C-reactive protein; ESR, erythrocyte sedimentation rate; CAR, CRP-to-albumin ratio; CTD, connective tissue disease.

### Cardiac involvement in this cohort

The incidence of cardiac involvement was 29.5% (31/105). Among these 31 cases, the male-to-female ratio was 16:15, with a mean age of 57.87 ± 12.06 years. The affected cardiac structures included the aorta, conduction system, myocardium, pericardium, and valves. The median interval from the first RP symptom to cardiac diagnosis among the 31 patients with cardiac involvement was 4 months (range, 1–72 months). Notably, all cases of cardiac involvement were diagnosed after the initial diagnosis of RP.

The most common manifestation was aortic root dilation, observed in 12 patients (11.4%). One of these patients, who also had concurrent aortic valve insufficiency, underwent Bentall surgery for symptomatic relief. Arrhythmias were present in nine patients (8.6%), with the following specific types: sinus bradycardia (*n* = 3), atrial premature beats (*n* = 2), complete right bundle branch block (*n* = 1), pre-excitation syndrome (*n* = 1), atrial fibrillation (*n* = 1), and supraventricular tachycardia (*n* = 1). Among these, the patient with supraventricular tachycardia achieved sinus rhythm restoration following methylprednisolone therapy. Pericardial effusion was found in six patients (5.7%) and responded well to steroid treatment. Eight patients (7.6%) were diagnosed with hypertrophic ventricular septum, in whom five were confirmed by CMR and accompanied by localized cardiac inflammation in the absence of other identifiable causes. Valvular insufficiency was observed in four patients (3.8%), involving the aortic valve in two cases and the mitral valve in the other two.

### Factors associated with cardiac involvement in RP patients

All patients with RP were divided into two groups according to the presence or absence of cardiac manifestations. Comparative analysis revealed no significant difference in the prevalence of CTD between the two groups (9.7 vs. 12.2%; *P* = 1.000). Similarly, no significant differences were observed in age (57.87 ± 12.06 vs. 54.61 ± 10.97 years; *P* = 0.180) or sex distribution (female/male: 15/16 vs. 36/38; *P* = 0.980). Traditional cardiovascular risk factors, including hypertension (29.0 vs. 14.9%; *P* = 0.092), diabetes mellitus (25.8 vs. 25.7%; *P* = 0.989), hyperlipidemia (32.3 vs. 28.4%; *P* = 0.691), smoking history (38.7 vs. 40.5%; *P* = 0.861), and alcohol consumption (16.1 vs. 18.9%; *P* = 0.735), also showed no statistically significant differences.

**Figure 1:**
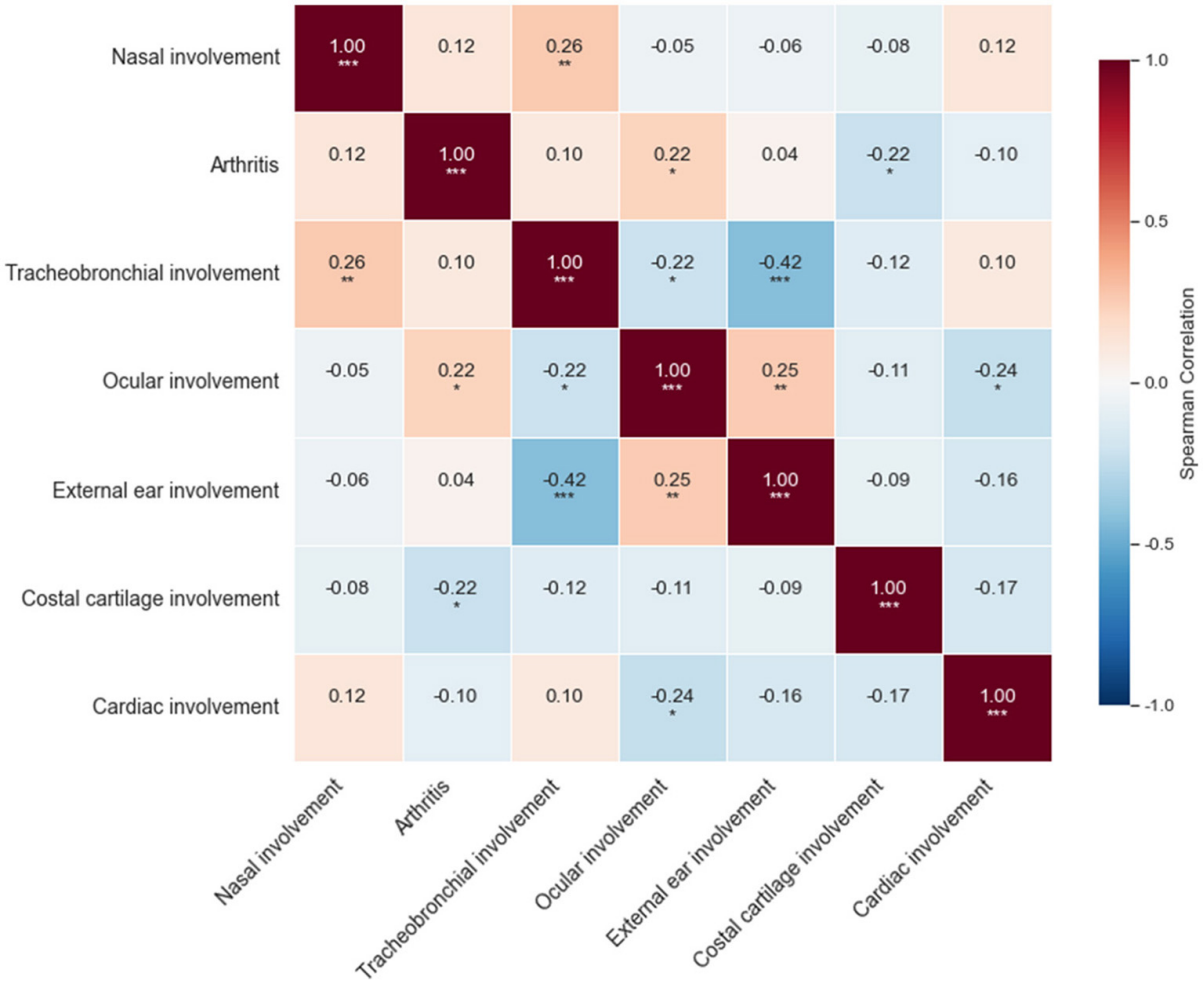
Correlations among various clinical manifestations (*P < 0.05; **P < 0.01; ***P < 0.001).

However, the proportion of ocular involvement was significantly lower in the cardiac involvement group than in the non-cardiac group (12.9 vs. 36.5%; *P* = 0.016). The incidence of costochondritis was also lower in patients with cardiac involvement, although this difference did not reach statistical significance (9.7 vs. 24.3%; *P* = 0.087). Regarding various clinical manifestations, a negative correlation was observed between cardiac involvement and ocular involvement (*r* = −0.24, *P* = 0.015) (Figure 1). Regarding laboratory parameters, patients with cardiac involvement exhibited significantly lower low-density lipoprotein (LDL) levels [2.60 (1.00, 4.37) vs. 2.84 (1.45, 5.30) mmol/L; *P* = 0.041], and LDL was negatively correlated with cardiac involvement (*r* = −0.20, *P* = 0.041). A trend toward lower total cholesterol (TC) levels was also observed in the cardiac involvement group, but the difference was not statistically significant [4.02 (2.23, 6.26) vs. 4.53 (2.99, 7.73) mmol/L; *P* = 0.051]. No other laboratory measures differed significantly between the groups, nor did they show any correlation with cardiac involvement. Detailed data are shown in Tables 2, 3.

**Table 2:** Comparison of clinical features in relapsing polychondritis: with vs. without cardiac involvement.

Feature	Cardiac involvement (*n* = 31)	Non- cardiac involvement (*n* = 74)	*P*-value
Age (ys)	57.87 ± 12.06	54.61 ± 10.97	0.180
Gender (F/M)	15/16	36/38	0.980
WBC (^*^10^9^/L)	7.94 (0.81, 26.09)	7.95 (3.59, 20.22)	0.555
NEU%	67.8 (23.5, 92.3)	70.2 (40.2, 96.0)	0.303
LY%	24.2 (0.9, 61.7)	22.2 (2.7, 47.9)	0.361
NLR	2.87 (0.38, 99.44)	3.18 (0.84, 35.56)	0.380
PLR	157.96 (53.02, 792.13)	160.10 (60.47, 1004.02)	0.877
TC (mmol/L)	4.02 (2.23, 6.26)	4.53 (2.99, 7.73)	0.051
LDL (mmol/L)	**2.60 (1.00, 4.37)**	**2.84 (1.45, 5.30)**	**0.041**
HDL (mmol/L)	1.23 (0.66, 2.18)	1.11 (0.57, 3.38)	0.382
TG (mmol/L)	0.96 (0.46, 2.40)	1.01 (0.37, 3.31)	0.250
ALB (g/L)	37.0 (27.8, 47.5)	37.2 (23.0, 53.8)	0.980
IgG (mg/dl)	1135.0 (656.0, 1930.0)	1210.0 (485.0, 2420.0)	0.787
IgA (mg/dl)	204.5 (90.3, 600.0)	268.0 (61.7, 634.0)	0.541
IgM (mg/dl)	74.8 (39.6, 300.0)	92.0 (27.7, 642.0)	0.492
C3 (mg/dl)	106.0 (47.6, 144.0)	112.0 (70.0, 182.0)	0.336
C4 (mg/dl)	24.5 (14.0, 50.0)	26.8 (11.0, 66.5)	0.275
CRP (mg/dl)	2.01 (0.16, 31.60)	1.69 (0.10, 23.10)	0.639
ESR (mm/h)	28.0 (4.0, 120.0)	29.0 (2.0, 100.0)	0.874
CAR	0.54 (0.04, 9.52)	0.51 (0.03, 7.35)	0.621
Concomitant CTD	9.7%	12.2%	1.000
Hypertension	29.0%	14.9%	0.092
Diabetes mellitus	25.8%	25.7%	0.989
Hyperlipidemia	32.3%	28.4%	0.691
Smoking history	38.7%	40.5%	0.861
Drinking history	16.1%	18.9%	0.735
Cutaneous lesions	12.9%	5.4%	0.231
Costal cartilage involvement	9.7%	24.3%	0.087
Ocular involvement	**12.9%**	**36.5%**	**0.016**
Arthritis	22.6%	32.4%	0.313
Nasal involvement	45.2%	32.4%	0.216
External ear involvement	38.7%	56.8%	0.091
Tracheobronchial involvement	87.1%	78.4%	0.299

WBC, white blood cell; NEU, neutrophil; LY, lymphocyte; NLR, neutrophil-to-lymphocyte ratio; PLR, platelet-to-lymphocyte ratio; TC, total cholesterol; LDL, low-density lipoprotein cholesterol; HDL, high-density lipoprotein cholesterol; TG, triglycerides; ALB, albumin; Ig, immunoglobulin; C3, complement 3; C4, complement 4; CRP, C-reactive protein; ESR, erythrocyte sedimentation rate; CAR, CRP-to-albumin ratio; CTD, connective tissue disease. Bold values indicate *p* < 0.05, considered statistically significant.

**Table 3:** Correlation between clinical results and cardiac involvement.

Clinical characteristics	R	*P*-value
Age (ys)	0.16	0.113
WBC (^*^109/L)	0.06	0.558
NEU%	−0.10	0.382
LY%	0.09	0.364
NLR	−0.09	0.382
PLR	−0.02	0.878
TC(mmol/L)	−0.19	0.05
LDL(mmol/L)	–**0.20**	**0.041**
HDL (mmol/L)	0.09	0.384
TG (mmol/L)	−0.11	0.252
ALB (g/L)	0.002	0.981
IgG (mg/dl)	−0.03	0.788
IgA (mg/dl)	−0.06	0.543
IgM (mg/dl)	−0.07	0.495
C3 (mg/dl)	−0.10	0.338
C4 (mg/dl)	−0.11	0.277
CRP (mg/dl)	0.05	0.641
ESR (mm/h)	0.02	0.875
CAR	0.05	0.624

WBC, white blood cell; NEU, neutrophil; LY, lymphocyte; NLR, neutrophil-to-lymphocyte ratio; PLR, platelet-to-lymphocyte ratio; TC, total cholesterol; LDL, low-density lipoprotein cholesterol; HDL, high-density lipoprotein cholesterol; TG, triglycerides; ALB, albumin; Ig, immunoglobulin; C3, complement 3; C4, complement 4; CRP, C-reactive protein; ESR, erythrocyte sedimentation rate; CAR, CRP-to-albumin ratio; CTD, connective tissue disease. Bold values indicate *p* < 0.05, considered statistically significant.

**Figure 2:**
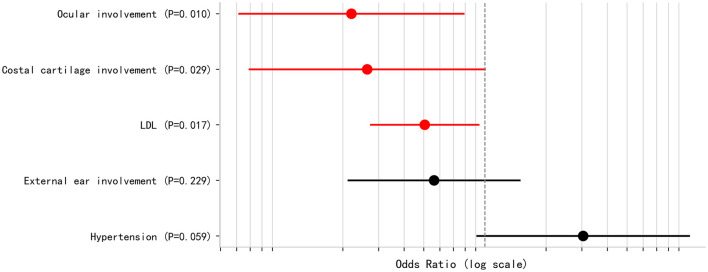
Firth logistic regression for cardiac involvement.

For Firth's logistic regression analysis, variables with *P* < 0.1 from the preceding analyses were considered candidate predictors. VIF analysis revealed a high collinearity (45.3) between LDL and TC. Therefore, the final model included LDL, ocular involvement, external ear involvement, costochondritis, and hypertension. The results showed that ocular involvement (OR: 0.22, 95% CI: 0.06–0.78; *P* = 0.010), costochondritis (OR: 0.26, 95% CI: 0.07–0.99; *P* = 0.029), and LDL level (OR: 0.51, 95% CI: 0.27–0.93; *P* = 0.017) were independent factors associated with cardiac involvement in RP (Figure 2).

**Figure 3:**
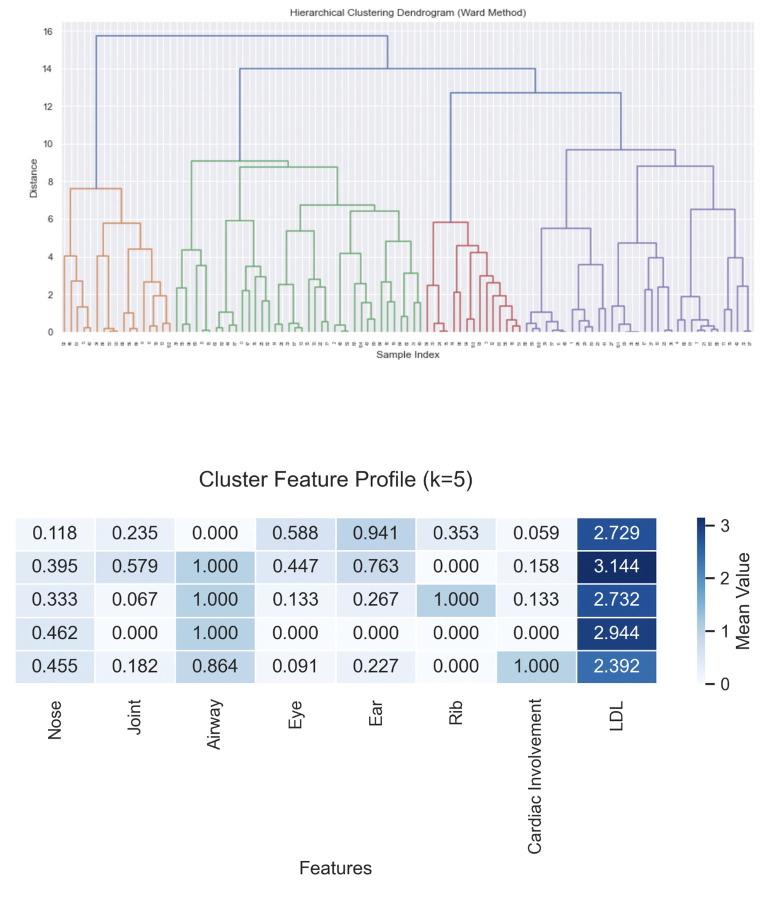
Hierarchical clustering and clinical characteristics of patients with relapsing polychondritis.

Using hierarchical clustering based on clinical manifestations and LDL levels, we stratified patients into five clusters as follows: Cluster 1 (ear-eye group), Cluster 2 (tracheobronchi-ear group), Cluster 3 (joint-tracheobronchi-costochondritis group), Cluster 4 (tracheobronchi only group), and Cluster 5 (tracheobronchi-cardiac group). Cluster 5 represented the cardiac-dominant group, in which all 22 patients had cardiovascular involvement, while the remaining nine patients with cardiac involvement were distributed across other clusters: Cluster 1 (*n* = 1), Cluster 2 (*n* = 6), and Cluster 3 (*n* = 2). Significant differences in organ involvement were observed among the clusters, with the exception of nasal involvement. Among these, patients in the cardiac-related cluster (Cluster 5) exhibited significantly lower LDL levels compared to the other clusters (Supplementary Table 1). Additionally, this cluster had lower proportions of ocular and costal cartilage involvement (Figure 3).

## Discussion

RP is a complex autoimmune disorder characterized by systemic organ involvement, primarily driven by immune-mediated mechanisms. The pathogenesis involves the recognition of cartilage-specific antigens, particularly Type II Collagen (CII) and matrilin-1. This autoimmune response promotes the release of pro-inflammatory cytokines and recruitment of immune cells to affected tissues, ultimately leading to cartilage destruction and systemic inflammation (9). In addition to cartilaginous tissue rich in CII, RP can also affect other proteoglycan-rich structures, such as heart valves and blood vessels. Although not the most common manifestations, cardiovascular complications constitute a critical dimension of RP management, with a reported prevalence varying considerably from 7.1 to 52% across studies (6, 10). This variability likely reflects heterogeneity in study populations and assessment methodologies. Despite this, the clinical significance of cardiovascular complications is substantial, as they are strongly associated with increased mortality (1).

In line with previous reports, our study found that cardiovascular involvement was common, observed in approximately 30% of the RP cohort, and presented a broad spectrum of disease manifestations. These included aortic root dilation, arrhythmias, cardiac effusion, valvular insufficiency, and localized myocarditis concurrent with septal thickening identified by CMR. Notably, effusive presentations like pericarditis responded favorably to steroid therapy.

Vascular involvement in RP has been reported in 5 to 42% of cases (11). The prevalence of aortic root dilatation in our cohort (11.4%) is higher than that reported in the French cohort (6.4%) (12), and is also greater than the incidence in a general middle-aged population in Italy (3.4%−7.3%) (13). One possible reason relates to differences in imaging techniques, as we used echocardiography, whereas the French study employed aortic CT. In addition, ethnic differences and disease heterogeneity, particularly the higher proportion of tracheobronchial involvement in our center, may also play a role. Tomelleri et al. further highlighted the aortic and branch vessel could be detected mainly at an advanced disease course (14). Notably, two patients were identified with concomitant small-vessel vasculitis. Although no obvious clinical vascular complications were observed in the patient with MAGIC syndrome in our cohort, Luo et al. have indicated this syndrome is associated with a higher prevalence of aortitis (15). Hypertension is a well-established risk factor for aortic dilation. According to Wu et al., the prevalence of aortic root dilation in hypertensive patients is 12.83% (16). Given this prevalence and the inclusion of only 20 hypertensive patients in our study, corresponding to an expected 2–3 cases, the observation of 12 cases in the entire cohort suggests that factors beyond hypertension likely contribute to this condition. Furthermore, the prevalence of aortic root dilation did not differ significantly between patients with and without hypertension (20.0 vs. 9.4%; *P* = 0.236, Supplementary Table 2). Nevertheless, hypertension remains a potential confounder affecting vascular remodeling, and its impact should be further clarified through long-term follow-up with larger sample sizes and sensitivity analyses. In clinical practice, physicians should maintain a low threshold for cardiovascular evaluation in RP patients who present with traditional cardiovascular risk factors.

Yamazaki et al. first reported a case of RP associated with aortic insufficiency as early as 1966 (17). Valvular involvement in RP mainly affected the aortic and mitral valves, consistent with our observations, which was likely attributable to inflammatory infiltration and fibrosis (18). Although most valvular lesions remain sub-clinical, some patients may develop severe heart failure symptoms necessitating surgical intervention (19). Moreover, postoperative complications such as valve dehiscence and periprosthetic leakage have been frequently reported.

The clinical significance of arrhythmias in RP should not be neglected, although it remains uncertain whether they represent a comorbidity or a direct complication of the disease. The prevalence of atrial fibrillation in our RP cohort (0.95%) is slightly lower than the reported prevalence in the Chinese adult population (1.6%) (20). However, our analysis excluded major causes of AF, such as thyroid dysfunction, coronary artery disease, electrolyte disturbances, congenital heart disease, and hereditary cardiomyopathies, which likely contributed to the lower observed rate. Moreover, arrhythmias in RP may be different from those in conventional disease. A potential underlying mechanism for arrhythmias may involve histopathological fibrosis of the atrioventricular node and the His-Purkinje conduction system (21). In support of this, de Carvalho et al. reported that glucocorticoid pulse therapy may reverse heart block and obviate the need for pacemaker implantation (22). Consistent with these findings, one patient with supraventricular tachycardia recovered following treatment with glucocorticoid and cyclophosphamide. Interventricular septal hypertrophy with concomitant cardiac inflammation was identified in five patients, in the absence of hypertension or other identifiable causes. Genetic testing or biopsy can be performed to further investigate the etiology. Although pericarditis is a common cardiac manifestation in CTDs like SLE, pSS, and RA (23), it was found less frequently in our study cohort, with a prevalence of 5.7%.

Electrocardiography, echocardiography, and CMR are essential tools for evaluating cardiac involvement in RP. However, whether angiography and/or positron emission tomography/computed tomography should be employed in routine clinical practice remains controversial. Endomyocardial biopsy may be considered in selected cases to confirm the diagnosis or to differentiate the etiology of myocarditis and other cardiac abnormalities.

Three patients in our cohort had both RP and associated CTDs: one with pSS and aortic regurgitation, one with pSS and premature atrial contractions, and one with AAV and mitral regurgitation. Corticosensitive cardiovascular manifestations, including pericardial effusion and supraventricular tachycardia, occurred only in patients without concomitant CTDs.

There is a notable scarcity of literature characterizing risk factors for cardiac involvement in RP to date. Contrary to some previous reports (10), our analysis revealed no significant associations with conventional demographic factors (age, gender) or traditional cardiovascular risk factors. Additionally, while Hou et al. identified neutrophil-to-lymphocyte ratio (NLR) > 6.41 as an independent risk factor for cardiac involvement and a marker of disease activity in RP (15, 24), our cohort showed no significant differences in inflammatory indices such as ESR, CRP, and NLR between patients with and without cardiac involvement. This discrepancy led us to hypothesize that localized cardiac inflammation may hold greater pathophysiological significance than systemic inflammation, and that its intensity may not be reliably reflected by circulating biomarkers. This hypothesis is strongly supported by our CMR findings, which directly visualized myocardial inflammation in a subset of patients, underscoring the value of this non-invasive tool for early diagnosis and prognosis assessment, as previously demonstrated in other systemic diseases (25, 26).

A pivotal and novel finding of our study was the inverse association with lower LDL levels, which appears to contradict conventional cardiovascular risk paradigms. We speculate this phenomenon may share a pathophysiological basis with the “lipid paradox” observed in other chronic inflammatory conditions such as RA (27). In this paradigm, lower cholesterol levels are linked to higher cardiovascular mortality, likely reflecting a state of heightened inflammatory activity and cachexia. We also observed positive correlations of TC and LDL with ALB, and negative correlations of TC with inflammatory markers (ESR, CRP, CAR) (Supplementary Table 3). Accordingly, we propose that lower LDL in RP may similarly reflect a more systemic inflammatory state, and that cardiac damage is primarily driven by inflammatory infiltration rather than atherosclerotic ischemia. Regrettably, detailed and reliable data on the timing, dosage, and duration of statin use, as well as glucocorticoid or immunosuppressive therapy at the exact time of lipid evaluation, were not consistently available in the medical records for all patients. Therefore, we were unable to include these medication data in the regression analysis.

In our study, patients with cardiac involvement showed a lower likelihood of ocular and costochondritis involvement. In contrast, Hou et al. reported that tracheobronchial and chest wall involvement were adverse factors for cardiac complications. Previous research has proposed subclassifying RP patients based on organ involvement patterns. For instance, cardiovascular manifestations have been shown to occur predominantly in the hematologic subtype, while being less common in the respiratory form (28). These discrepancies reflected the considerable heterogeneity among RP patients and across study cohorts. Of note, our respiratory center is a leading discipline at our institution and serves a large patient population. Consequently, airway involvement was more frequently observed in our cohort, which may influence disease classification.

This study has several limitations. First, the assessment of long-term cardiac outcomes in RP requires longitudinal follow-up. Second, the sample size of this study was relatively limited, which precluded comprehensive multivariable adjustments or subgroup analyses for traditional cardiovascular risk factors such as hypertension, as well as medication use (statins, glucocorticoids, and immunosuppressive agents) at the time of lipid evaluation, to fully exclude their confounding effects. Third, studies are needed to identify and validate biomarkers for specific cardiac manifestations, which is essential for advancing precision medicine. Fourth, hierarchical clustering, as an exploratory unsupervised learning approach, may yield cohort-dependent results; therefore, additional clustering methods on larger datasets are required. Finally, further research into the underlying immune mechanisms of cardiac involvement is necessary to inform more effective therapies.

## Conclusion

In conclusion, cardiovascular involvement in RP is common and manifests as a broad spectrum of clinical presentations. Our study identifies the absence of ocular involvement and costochondritis, along with lower LDL levels, as factors associated with cardiac disease in RP. These findings underscore the distinct phenotype of RP with cardiac involvement and highlight potential clinical indicators for its identification.

## Data Availability

The datasets presented in this article are not readily available because the datasets generated and/or analyzed during the current study are available from the corresponding author upon reasonable request. Requests to access the datasets should be directed to shty.1229@163.com.
