# Supplementary material for: Clinical characteristics and associated factors of cardiovascular involvement in patients with relapsing polychondritis
Source: Front Med (Lausanne). 2026 Jul 31;13:1877659. doi: 10.3389/fmed.2026.1877659 (PMC13472794; doi:10.3389/fmed.2026.1877659)
Supplement: Supplementary file 1 [file Table_1.docx]

**Supplementary table 1. Comparison of clinical features across hierarchical clusters**

| **Cluster**  **(n)** | **Nose**  **(%)** | **Joint**  **(%)** | **Tracheobronchi**  **(%)** | **Eye**  **(%)** | **Ear**  **(%)** | **Costochondritis**  **(%)** | **Cardiac**  **(%)** | **LDL**  **(mmol/L)** |
| --- | --- | --- | --- | --- | --- | --- | --- | --- |
| **1 (17)** | 0.12 | 0.24 | 0 | 0.59 | 0.94 | 0.35 | 0.06 | 2.73 |
| **2 (38)** | 0.40 | 0.58 | 1 | 0.45 | 0.76 | 0 | 0.16 | 3.14 |
| **3 (15)** | 0.33 | 0.07 | 1 | 0.13 | 0.27 | 1 | 0.13 | 2.73 |
| **4 (13)** | 0.46 | 0 | 1 | 0 | 0 | 0 | 0 | 2.94 |
| **5 (22)** | 0.45 | 0.18 | 0.86 | 0.09 | 0.23 | 0 | 1 | 2.39 |
| **P-value** | 0.199 | < 0.001 | < 0.001 | < 0.001 | < 0.001 | < 0.001 | < 0.001 | 0.014* |

*** Cluster 5 vs. Non-Cluster 5**
