# Supplementary material for: Clinical characteristics and associated factors of cardiovascular involvement in patients with relapsing polychondritis
Source: Front Med (Lausanne). 2026 Jul 31;13:1877659. doi: 10.3389/fmed.2026.1877659 (PMC13472794; doi:10.3389/fmed.2026.1877659)
Supplement: Supplementary file 2 [file Table_2.docx]

**Supplementary Table 2. Comparison of aortic root dilation in relapsing polychondritis: **With versus Without hypertension****

| ****Feature**** | **Hypertension**  **(n = 20)** | ****Non- hypertension****  **(n = 85)** | ***P***-value**** |
| --- | --- | --- | --- |
| **aortic root dilation** | 20.0% | 9.4% | 0.236 |

Note: Abbreviations as in Tables 1.
