# Supplementary material for: Clinical characteristics and associated factors of cardiovascular involvement in patients with relapsing polychondritis
Source: Front Med (Lausanne). 2026 Jul 31;13:1877659. doi: 10.3389/fmed.2026.1877659 (PMC13472794; doi:10.3389/fmed.2026.1877659)
Supplement: Supplementary file 3 [file Table_3.docx]

**Supplementary table 3. Correlation between LDL/TC and Clinical Parameters**

| **Clinical characteristics** | **LDL** | | **TC** | |
| --- | --- | --- | --- | --- |
|  | **r** | ***P*-value** | **r** | ***P*-value** |
| WBC (*109/L) | 0.01 | 0.930 | 0.003 | 0.973 |
| NLR | -0.11 | 0.257 | -0.05 | 0.598 |
| PLR | -0.11 | 0.289 | -0.13 | 0.183 |
| IgG (mg/dl) | 0.17 | 0.083 | **-0.22** | **0.027** |
| IgA (mg/dl) | -0.02 | 0.864 | **-0.24** | **0.019** |
| IgM (mg/dl) | 0.05 | 0.640 | 0.02 | 0.815 |
| C3 (mg/dl) | 0.17 | 0.100 | -0.08 | 0.433 |
| C4 (mg/dl) | 0.10 | 0.328 | -0.06 | 0.556 |
| CRP (mg/dl) | -0.08 | 0.427 | **-0.26** | **0.009** |
| ESR (mm/h) | -0.02 | 0.808 | **-0.20** | **0.048** |
| CAR | -0.11 | 0.276 | **-0.28** | **0.004** |
| ALB | **0.31** | **0.002** | **0.34** | **< 0.001** |

Note: Abbreviations as in Tables 1.
